# First record of *Biromiris* from the Oriental region, with a new species from Philippines (Hemiptera, Heteroptera, Miridae, Phylinae)

**DOI:** 10.3897/zookeys.677.11308

**Published:** 2017-05-29

**Authors:** Ram Keshari Duwal, Tomohide Yasunaga, Toshiya Hirowatari

**Affiliations:** 1 Entomological Laboratory, Faculty of Agriculture, Kyushu University, Fukuoka, 812-8581, Japan; 2 Research Associate, Division of Invertebrate Zoology, American Museum of Natural History, New York, NY 10024, USA

**Keywords:** *Biromiris tomokunii*, Leucophoropterini, Miridae, new species, Philippines, Phylinae, taxonomy

## Abstract

The unique genus *Biromiris* Schuh is recognized from the Oriental region for the first time, with the description of a new species, *Biromiris tomokunii* **sp. n.**, from the Philippines. The new species is documented with photographic images of the dorsal habitus and male genital structures. A key to all known species of *Biromiris* is provided.

## Introduction

*Biromiris* Schuh, 1984 (Phylinae), containing six described species from the Australian region, forms one of the smaller and rarely encountered genera of the plant bug tribe Leucophoropterini (Schuh 1984, Menard and Schuh 2011). The present work documents a new species of this unique genus from Palawan Island of the Philippines, and represents the first record for *Biromiris* from the Oriental region. A key is provided to facilitate identification of all the species of *Biromiris*.

## Materials and methods

The observed specimen (holotype) is deposited in the Department of Zoology, National Museum of Nature and Science, Tsukuba, Japan (NSMT). A Data Matrix code label, which uniquely identifies the specimen and is referred to as ‘unique specimen identifier’ (USI), was attached to the holotype. The code (e.g., AMNH_PBI 00380531) was digitized on the Arthropod Easy Capture (formerly the Planetary Biodiversity Inventory) database maintained by the American Museum of Natural History, New York, USA (http://research.amnh.org/pbi/heteropteraspeciespage/speciesdetails.php?fromall=fromall&speciesid=89593&genusid=5768).

All measurements are given in millimeters. The terminology mainly follows Menard and Schuh (2011) and Schuh (1984). Observation and measurements were performed under Olympus SZX7, dorsal images were taken in Leica S8APO equipped with Leica 10445930 1.0×, attached to Canon EOS Kiss digital camera body, and genital structures were made with Nikon ECLIPSE E400. The key to species was principally based on descriptions by Menard and Schuh (2011) and Schuh (1984).

## Results

### Biromiris

Taxon classificationAnimaliaHemipteraMiridae

Schuh, 1984

Biromiris Schuh, 1984: 206, type species by original designation: Biromiris enarotadiSchuh 1984; Schuh 1995: 241 (cat.), 2002–2014 (http://research.amnh.org/pbi/catalog/), Menard and Schuh 2011: 74 (diag., re-descr.)

#### Diagnosis.

Distinguished by carinate lateral margin of pronotum; transverse roll (or, double chin) gula; terete or slender antennal segments III & IV; white dorsolateral area on metepisternum dorsal to scent gland; partial transverse fascia on anterior corium; and form of male genital structures. For detailed descriptions see Schuh (1984: 206) and Menard and Schuh (2011: 74).

#### Distribution.

The Oriental to Australian regions across Wallacea.

#### Key to identification of species of *Biromiris*

**Table:** 

1	Antennal segments III and IV terete	**4**
–	Antennal segments III and IV slender (or, linear)	**2**
2	Body distinctly small, shorter than 2.30 mm; entire procoxa pale, tinged with red on distal region; cuneus with small white spot at inner corner; Philippines (Palawan)	***B. tomokunii* sp. n.**
–	Body size longer than 3.00 mm; procoxa dark reddish, with white distal margin; cuneus with wide white area on anterior margin	**3**
3	Body including ventral side basically chestnut; narrow vertex and pronotal carina; Australia (New South Wales)	***B. cassisi* Menard & Schuh**
–	Body including ventral side basically brown; wide vertex and broad pronotal carina; Australia (Queensland)	***B. binjour* Menard & Schuh**
4	Head, pronotum and scutellum orange brown or brown; antennal segment II entirely brown or basally golden	**5**
–	Head, pronotum and scutellum mahogany or chestnut; antennal segment II basally pale or mahogany and distally chestnut	**6**
5	Scent gland evaporatory area unicolorous with thoracic pleuron; pro- and mesocoxae and femora golden, and metafemora dark red; labium reaching apex of mesocoxa; Indonesia (West Irian), Australia (Queensland)	***B. enarotadi* Schuh**
–	Scent gland evaporatory area paler than thorax; all coxae and metafemora dark brown, and pro- and mesofemora pale; labium reaching apex of metacoxa; Australia (New South Wales)	***B. scheyville* Menard & Schuh**
6	Ventral side of head and thorax mahogany or chestnut; posterior margin of vertex concave; metatibia without spines; Papua New Guinea (Morobe), Australia (Queensland)	***B. bulolo* Schuh**
–	Ventral side of head and thorax golden brown; posterior margin of vertex straight; metatibia with suberect pale spines, Indonesia (West Irian)	***B. cyclops* Schuh**

### Biromiris tomokunii sp. n.

Taxon classificationAnimaliaHemipteraMiridae

http://zoobank.org/EB5D9D50-94EF-4424-A14D-41E56B3AC911

Figures 1 , 2

#### Type material.

Holotype male. PHILIPPINES, Palawan, Matalangao, 10.33°N, 119.25°E, 450m, 29.viii.1985, M. Tomokuni (NSMT) (AMNH_PBI 00380531).

#### Diagnosis.

**Figure 1.**
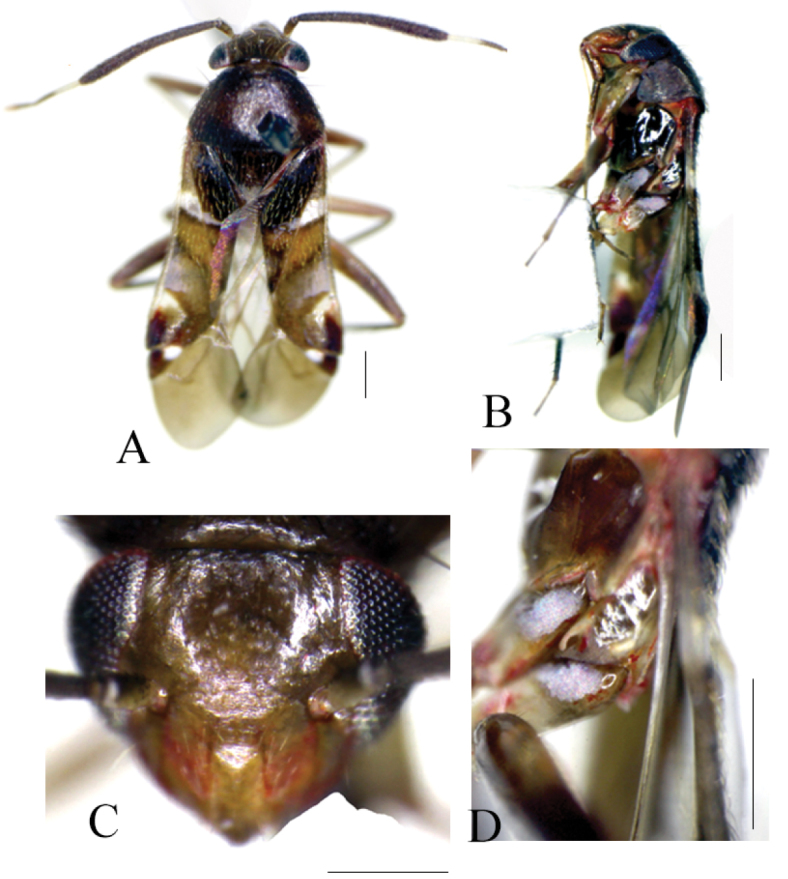
Habitus images of *Biromiris tomokunii*, holotype male. **A** dorsal view **B** lateral view **C** Head in frontal view **D** Scent gland evaporatory area. Scale bars, 0.5 mm.

**Figure 2.**
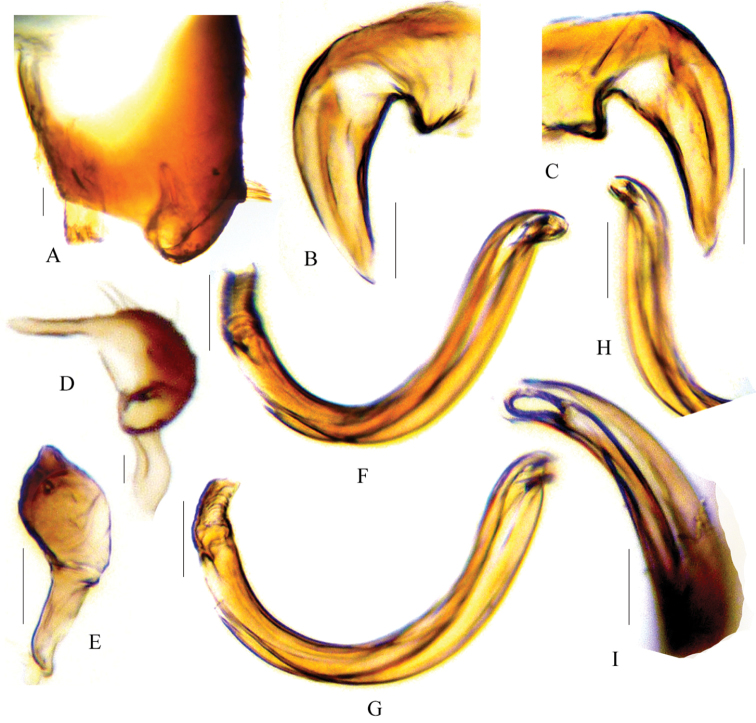
Male genital structures of *Biromiris tomokunii*. **A** Pygophore, right lateral view **B–C** Phallotheca **D** Left paramere, lateral view **E** Right paramere, anterior view **F–G** Endosoma **H–I** Apex of endosoma. Scale bars, **A–H** 0.2 mm; **I** 0.1mm.

Recognized by small size; brownish general coloration; wide vertex; slender (not terete) antennal segments III and IV; dark bunch of suberect setae on apex of the clavus; white transverse fascia and/or macula on the anterior and posterior corium; prominent spot on the inner corner of cuneus (Fig. 1); and unique form of male genital structures (Fig. 2).

The new species is distinguished from all congeners by its small size; slender antennal segments III and IV; anterior transverse white fascia continuous on clavus but narrow and not reaching claval commissure; distinct white macula on posterior corium at level of apex of clavus; and prominent white spot on inner corner of cuneus (Fig. 1A).

#### Description.

**Male**. **Coloration** (Fig. 1A–D): Body including dorsum basically brown. *Head*: yellowish brown with dark basal margin on vertex; mandibular and maxillary plates, and ventral side of head tinged with red; clypeus same coloration as frons, with dark apex. *Antenna*: dark brown, with pale base on segment I and white on basal ½ of segment III. *Labium*: yellowish, tinged with red on segment I, apical segment darker. *Thorax*: pronotum and scutellum dark brown; thoracic pleura brown with ivory or white surface on metepisternum dorsal laterally to scent gland evaporatory area (Fig. 1D); peritreme of scent gland evaporatory area white and dark (Fig. 1D). *Legs*: entire procoxa, distal half of meso- and metacoxae pale, extreme apices tinged with red, and remaining proximal regions brown; all trochanters pale (or white); all femora yellowish, except for brown distal half of hind femur; entire protibia, proximal half of mesotibia and extreme base and apical 1/5 region of metatibia pale, and remaining regions dark; tarsal segments pale. *Hemelytron*: brown with dark brown on anterior regions of clavus and corium, and on apex of the clavus; anterior corium with distinctly white transverse fascia margined with dark posteriorly, reaching middle of clavus (Fig. 1A); posterior corium with white fascia at about apex of radius; posterior lateral region of corium red; cuneus brown, laterally tinged with red, and with prominent white spot on inner corner of cuneus; membrane grayish brown.

**Surface and vestiture**: *Head*: shiny and weakly shagreen; dorsally covered with pale or yellowish semi-erect simple setae, and ventrally with dark erect setae. *Antenna*: covered with appressed pale setae. *Thorax*: pronotum and scutellum weakly shagreen and impunctate; pronotum uniformly distributed with dark or black semi-erect setae. *Hemelytron*: weakly shagreen and impunctate with several reflecting patches; corium with mixed vestiture, semi-erect black and yellow or golden simple setae; clavus with dark erect setae at the apex; cuneus with dark setae only. *Legs*: all legs covered with appressed pale or dark setae; and hind tibia with two rows of sub-erect dark spines. STRUCTURE: macropterous, body elongate-oval. *Head*: triangular, clypeus barely observed from dorsal view; vertex weakly concave, with carinate basal margin; eyes relatively small. *Antenna*: antennal fossae not continuous with inner margin of eyes, segment II clavate and relatively thick, and segments III and IV not terete. *Labium*: reaching apex of metacoxae. *Thorax*: pronotum convex and trapezoidal, with narrow collar-like margin lies underneath vertex, and lateral sides of the anterior pronotum with narrow carina; mesoscutum obscurely exposed; scent gland evaporatory area more or less triangular, with distinctly elevated peritreme. *Hemelytron*: posterior corium (anterior to cuneus) splayed out; cuneal fracture distinctly incised; cuneus small and triangular. *Legs*: all femora long and sub-parallel except for narrow extreme apex; tarsal segment I and II sub-equal, and segment III relatively longer. GENITALIA (Fig. 2A–I): *Pygophore*: trapezoidal, with bunch of stiff bristles sub-apically on ventral side (Fig. 2A). *Left paramere*: small, with elongated posterior process, and distinctly short and narrow anterior process (Fig. 2D). *Right paramere*: leaf like, with a short and blunt apical process (Fig. 2E). *Phallotheca*: wide base and narrow apex as in Figure 2B–C. *Endosoma*: simple, weakly S-shaped, with small weakly sclerotized apically placed secondary gonopore (Fig. 1F–I).

***Female***. Unknown.

**Measurements**: 1♂: Total body length 2.21; length from apex of clypeus to cuneal fracture 1.90; width of head across eyes 0.51; width of vertex 0.28; length of antennal segments I–IV ? (broken); basal width of pronotum 0.70; length of pronotum 0.42; width across hemelytron 0.78; length of metafemur, tibia and tarsus 0.74, 1.14, 0.20.

#### Etymology.

Named after Dr. Massaki Tomokuni (Curator Emeritus, Department of Zoology, NSMT), collector of the specimen.

#### Distribution.

Philippines (Palawan).

## Discussion

All six species of *Biromiris* described by Schuh (1984) and Menard and Schuh (2011) were based mainly on external morphology. The male and female genitalia are not examined for any species other than type species, *Biromiris enarotadi* Schuh; the male genital structures were illustrated and described (Schuh 1984). In this study the male genitalia of the holotype was examined in detail (Fig. 2).

The majority of *Biromiris* species are represented by just a few specimens or the holotype only; further researchers are encouraged to utilize broader surveys to clarify the zoogeographical distribution pattern and biology of this genus (Schuh 1984, Menard and Schuh 2011). *Biromiris* was previously known only from the tropical Australian region including New Guinea (Menard and Schuh 2011, Schuh 1984). The present discovery of the new species, *B. tomokunii* from the Philippines suggests that the genus is perhaps more widely distributed in the Oriental region and will probably include Sundaland elements as evidenced by occurrence of the new species on Palawan Island. Additionally, we anticipate more species of *Biromiris* will be found to occur widely across Wallacea, as revealed for quite a few phyline genera (Schuh 1984) and some of Bryocorinae (cf. Namyatova et al. 2016, Yasunaga and Ishikawa 2016).

Almost nothing is known about the biology of *Biromiris*, as most of species were collected in different kinds of trap (e.g. light traps and pitfall traps) except for *B. scheyville* Menard & Schuh; where the host plant was confirmed as Myrtaceae (Menard & Schuh 2011). The biology of the present new species, *B. tomokunii* remains unknown.

## Supplementary Material

XML Treatment for Biromiris

XML Treatment for Biromiris tomokunii
